# Autoimmune Thyroid Disorders Are More Prevalent in Patients with Celiac Disease: A Retrospective Case-Control Study

**DOI:** 10.3390/jcm11206027

**Published:** 2022-10-12

**Authors:** Maria Pina Dore, Giuseppe Fanciulli, Malik Rouatbi, Sandro Mereu, Giovanni Mario Pes

**Affiliations:** 1 Dipartimento di Medicina, Chirurgia e Farmacia, Clinica Medica, University of Sassari, Viale San Pietro 8, 07100 Sassari, Italy; 2 Baylor College of Medicine, One Baylor Plaza Blvd. Houston, Houston, TX 77030, USA; 3 Dipartimento di Medicina, Chirurgia e Farmacia, Endocrine Unit, AOU Sassari, University of Sassari, Viale San Pietro 43b, 07100 Sassari, Italy

**Keywords:** celiac disease, autoimmune thyroid disease, case-control study, Sardinia

## Abstract

Background. Among patients with celiac disease (CD), there is an increased incidence of autoimmune thyroid disorders (AITDs), with hypothyroidism being more frequent than hyperthyroidism. This retrospective case-control study aimed to explore the prevalence of TDs in a population of adult celiac patients from Northern Sardinia, a geographic area with a high prevalence of autoimmune disorders. Methods. Data were collected from consecutive patients with CD (cases) and without CD (controls) who were undergoing upper endoscopy and referred to a tertiary gastroenterology section of a teaching hospital (University of Sassari, Italy). Thyroid disorders were stratified as (i) autoimmune: including Hashimoto’s disease in euthyroidism or with hypofunction, and Graves’ disease; or (ii) non-autoimmune: thyroid nodules/goiter, iatrogenic thyroid hypo/hyperfunction, and thyroidectomy for any reason, including cancer. Results. Among a total of 8489 participants (females 5839, 64.7%) enrolled, there were 623 (7.3%) celiac patients and 7866 controls (92.7%). The overall frequency of TDs was 1177 (13.9%) and was higher (26.0%) in celiac patients than in controls (12.9%) (*p* < 0.001). The difference between AITDs (15.4% vs. 7.5%) and no-AITDs (2.7% vs. 1.1%) was statistically significant in CD patients compared with controls, respectively, and prevailed in the fifth and sixth decades. Hashimoto’s thyroiditis was more commonly associated with gland hypofunction. Odds ratios with their 95% confidence intervals (CIs) for the presence of AITDs were calculated, adjusting for sex, age, body mass index, smoking habits, occupation, and residence, and they were 2.387 (95% CI 1.857–3.068, *p* < 0.001) in CD patients, 5.855 (95% CI 4.434–7.731, *p* < 0.001) for female sex, and 1.012 (95% CI, 1.007–1.017, *p* < 0.001) for age. Conclusion. These results suggest the need for surveillance for TDs in patients with CD at onset and during follow-up.

## 1. Introduction

Celiac disease (CD) occurs upon exposure to dietary gluten in genetically predisposed individuals. Whereas predisposing genes and ingestion of gluten are both pivotal to the development of CD, most HLA-DQ2/DQ8 carriers (about 30% of the general population) who are exposed to gluten (>99% of the world population) do not develop CD [1]. Among susceptible individuals, indeed, only 1% to 3% [2,3] will develop CD, therefore indicating the importance of additional genetic and/or environmental factors other than gluten in the determinism of CD [4,5,6,7]. In particular, factors able to influence the intestinal environment early in life, such as breastfeeding, infections, and alterations of gut microbiota have gained a lot of interest [6]. In patients with CD, malabsorption is the consequence of the enteropathy characterized by mucosal inflammation, crypt hyperplasia, and ultimately, villous atrophy.

In the past, CD was considered a rare disorder, mostly affecting individuals of European origin, and usually characterized by onset during the first years of life.

At that time, the diagnosis was entirely based on the detection of typical gastro-intestinal symptoms and confirmation by a small intestinal biopsy obtained by a troubled and time-consuming procedure using a metallic capsule, the “Crosby–Kugler capsule” [8].

The availability of highly sensitive and specific serological tests, such as the anti-endomysium (EMA), the anti-TG (t-TG), and the anti-deamidated gliadin antibodies (Ab) tests, allowed for the better evaluation of the true prevalence of CD, revealing an unsuspected frequency of clinically atypical or even silent forms. As an example, a study performed in Italy reported that asymptomatic cases outnumbered symptomatic cases by a ratio of 7:1 [9]. A meta-analysis including 275,818 individuals found that the pooled global prevalence of CD was 1.4%, based on tTG-Ab and/or EMA-Ab, and 0.7% by biopsy in 138,792 individuals, and it was more common in females and in children [10].

The highest prevalence of CD in the world has been reported among Saharawi (5.6%), an African population of Arab-Berber descent, originally living in Western Sahara. According to the Italian Istituto Superiore della Sanità (https://www.iss.it/en/web/guest, accessed on 29 May 2022, it has been calculated that, in the Italian population, the expected number of CD patients was around 600,000. However, only 233,147 of them (66% females) had been diagnosed up to that date. Noteworthily, regional variations in the prevalence have been observed. For example, Meloni et al. detected a prevalence of 1.43% in children from Northern Sardinia [11].

The clinical presentation of CD nowadays varies greatly. Less than 50% of adults present with gastrointestinal symptoms, and patients can complain of a spectrum of extra intestinal symptoms [6,12,13,14]. Moreover, CD can be closely associated with a number of autoimmune disorders (AIDs), such as type 1 diabetes mellitus and autoimmune thyroid disorders (AITDs), including Hashimoto’s thyroiditis (HT) and Graves’ disease (GD) [15]. Additional AIDs, such as neurological disorders and atopic disorders, among others, have also been observed in patients with CD [16,17]. The co-occurrence of CD with other AIDs further corroborates a shared genetic susceptibility, especially related to the HLA system [18,19,20].

HT is, in general, the most common AITD, with an incidence of about 1 case per 1000 persons per year [21], and it is the most frequent endocrine disorder [22], with a clear predominance in females. HT is a chronic inflammation in which the pathologic features of lymphocytic infiltration, especially of T cells, and follicular destruction are the histological hallmarks, being the leading cause of hypothyroidism in the iodine-sufficient areas of the world [23].

In a meta-analysis including 15,629 CD cases and 79,342 controls, Sun et al. detected that an overall prevalence of thyroid disorders (TDs) significantly increased in patients with CD compared with the control group (OR 3.08, *p* < 0.001), without difference between gluten-treated and untreated groups [24]. For these reasons, current international guidelines suggest CD screening in first-degree relatives of patients with autoimmune disorders such as type 1 diabetes and thyroid disease [25].

Sardinia is an Italian region whose population is characterized by a homogenous genetic background and an exceptionally high prevalence of AIDs, including type 1 diabetes, autoimmune thyroiditis, and CD [26]. Additionally, patients with CD, especially those not compliant with a strict gluten-free diet (GFD) show an increased risk of developing additional AIDs [13,15,27,28]. In a previous study involving a pediatric population from Sardinia, a strong association between AITDs and CD was observed [29].

The aim of this study was to evaluate the prevalence of TD in adult patients with CD in a defined geographical area with a high frequency of both disorders.

## 2. Materials and Methods

This was a retrospective case-control study performed in a tertiary referral gastroenterology section of a teaching hospital (University of Sassari, Italy) in subjects undergoing upper endoscopy. Biopsy-confirmed CD patients were labeled as “cases”, and the remaining scoped patients without CD as “controls”. The outcome of the study was to estimate the frequency of thyroid disorders (TDs) in patients with CD compared to subjects without CD, according to the variables included in the study.

### 2.1. Patients Eligibility

Patients older than 18 years, with a detailed clinical history and full information about current treatment, were recruited for the study. Missing information about present/absent TDs, taken medicines, anamnesis, and/or demographics were considered exclusion criteria.

### 2.2. Data Collection

Personal data, including sex, age, residence, occupation, smoking habit, and anthropometric measures, were retrieved from the charts of patients who underwent upper endoscopy for any reason. Before the procedure, the patient was interviewed by a gastroenterology fellow and supervised by the same attending gastroenterologist for the whole study period, using a chart structured in an equal manner across the entire studied cohort, as previously described [30]. In addition, detailed information was collected for the diagnosis of CD, any diagnosed TD, and all taken medications. For each patient, only data from the index procedure were included in the analysis.

### 2.3. Diagnosis

**Figure 1:**
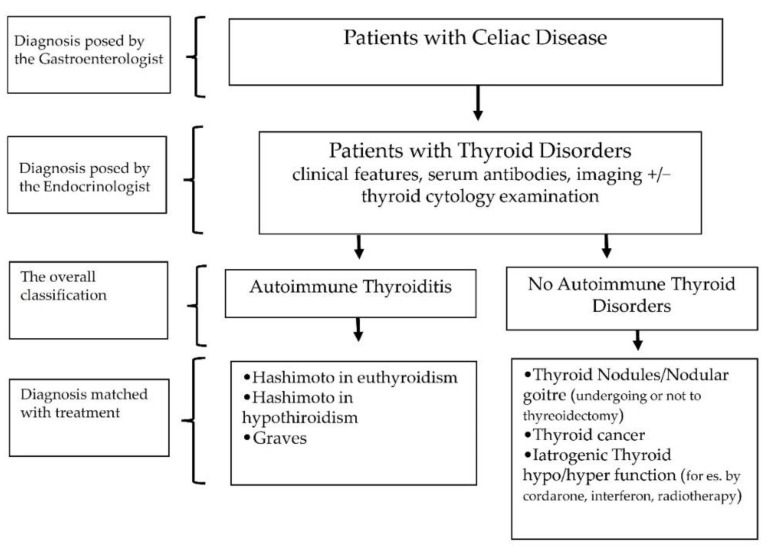
Diagnosis of thyroid disorders in study participants.

The diagnoses of CD and TDs were posed by the respective specialists according to national and international guidelines/expert consensus developed during the time (Figure 1). On the basis of clinical features, presence of serum antibodies against thyroperoxidase and thyroglobulin, thyroid imaging, and, when required, examination of thyroid cytology, TDs were classified into autoimmune (AITDs) and non-AITDs (Figure 1).

In the first group (AITDs), subjects were categorized as follows: (i) HT in the euthyroid state; (ii) HT in the hypothyroid state; and (iii) Graves’ disease. In the second group (non-AITD), subjects were subdivided into the following groups: (i) solitary thyroid nodule and multinodular goiter; (ii) thyroid cancer, and (iii) drug-induced (i.e., amiodarone, anticancer drugs, lithium, interferons, etc.) thyroid dysfunction (Figure 1). The diagnosis of functional TDs was additionally confirmed by matching the TD with treatment with levothyroxine or methimazole accordingly.

### 2.4. Ethical Approval

The study was approved by the local ethics committee of “Azienda Ospedaliero Universitaria di Sassari” (Prot. N° 2021/CE, 2018).

### 2.5. Statistical Analysis

Categorical variables were expressed as numbers and percentages. For scalar variables, the mean and standard deviation were calculated. The association between AITDs and CD was determined using univariable and multivariable logistic regression models and unadjusted and adjusted odds ratios (ORs), and the 95% CI was calculated according to the covariates taken into account. Age was grouped by decades, and residence was dichotomized into urban and rural. Occupation was stratified from the highest (Class I) to the lowest (Class IV) in four classes, as previously reported [31]. In relation to smoking habit, patients were labeled as never, or former and current smokers. The body mass index (BMI) was calculated by using the formula weight (kg)/height (m)^2^, and obesity was defined as a BMI ≥ 30 kg/m^2^. All statistical analyses were performed using SPSS statistical software (version 22.0, Chicago, IL, USA). *p*-values lower than 0.05 were considered statistically significant.

## 3. Results

In our cohort of 8489 (5839 females, 64.7%) patients, 1177 (13.9%) were affected by TDs, and as expected, the prevalence was statistically higher in females compared to males (19.2% vs. 4.1%), with a tendency to increase in the fifth and sixth decades (Table 1).

The occurrence of TDs was not influenced by the patient’s residence; instead, TDs were more frequent in patients belonging to the lowest occupation level (Class IV 17.0% vs. Class I 14.3%; *p* < 0.001) (Table 1). Interestingly, the different classes of BMI, as well as those of smoking habit, did not show any statistically significant relation to TDs. As expected, the prevalence of TDs was doubled in patients with CD compared with patients without CD, and the difference was statistically significant (26.0% vs. 12.9%) (Table 1). More specifically, among the 1177 patients with TDs, 162 were celiac patients.

Table 2 lists the specific TDs according to the presence of CD. The risk of AITD was increased in patients with CD compared with patients without CD, and in both cases (euthyroidism and hypothyroidism), the difference was statistically significant (2.7% vs. 1.1% and 11.4% vs. 5.6%, respectively) (Table 2). Similarly, a greater number of patients with CD were affected by Graves’ disease, albeit data were not statistically significant (Table 2).

Moreover, TDs unrelated to autoimmunity were also more represented among CD patients although the small numbers did not allow for reliable conclusions (Table 2). Thyroidectomy was an exception, with a total of 314 (3.7%) procedures, and thus more frequent in CD patients compared with controls (9.5% vs. 3.2%, *p* < 0.001). The majority of patients without a thyroid gland underwent thyroidectomy according to the endocrinologist’s indication for the presence of multinodular goiter, large or suspicious nodules, and in a few cases, cancer (mostly papillary; Hurthle cell cancer in only one case).

**Figure 2:**
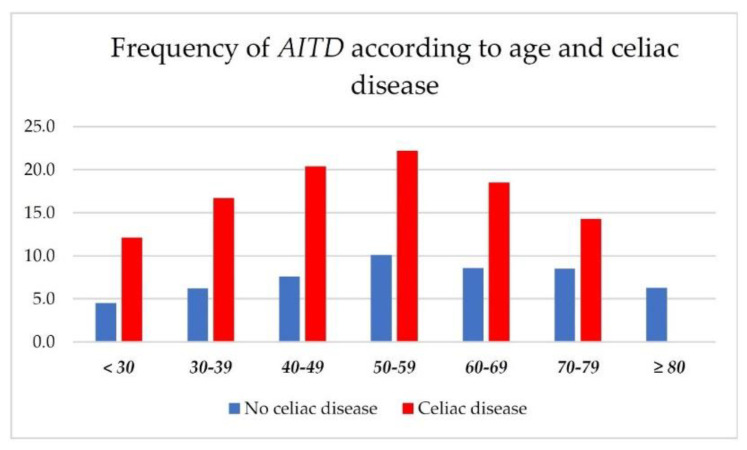
Frequency of autoimmune thyroid disorders (AITD) according to age (by decade) and presence of celiac disease.

In Figure 2 are represented the percentage of AITDs, and, similar to the overall TDs, they were more common in the fifth and sixth decades.

The results of univariable and multivariable logistic regression analysis, using the presence or absence of AITDs as the outcome according to CD, are shown in Table 3.

As expected, after adjusting for all covariates, the higher statistically significant adjusted ORs for AITDs persisted for the female sex (5.855; *p* < 0.001) and older age (1.012, *p* < 0.001), and the effect of occupation disappeared (Table 3). A strong association was confirmed between AITDs and CD, with significantly higher odds in the unadjusted (OR 2.382) and adjusted models (OR 2.387). Of note, additional subanalysis showed that the risk of AITDs in celiac disease was higher in males (10.254, 95% CI 4.390–23.95) than in females (1.856, 95% CI 1.326–2.596).

## 4. Discussion

Sardinia is a geographic area with an increased prevalence of autoimmune diseases, including type 1 diabetes, HT, and CD, among others [15,26,32]. More specifically, in this ancient genetic isolate, the distribution of HLA class II alleles and haplotypes differs from that of other Caucasian populations [33], with a very high frequency of CD-predisposing HLA class II DQ haplotypes [34]. Common pathogenic mechanisms may potentially drive more AIDs to occur in an individual patient [15]. In fact, CD and several autoimmune disorders share a common genetic predisposition, i.e., HLA-DQ2 or DQ8 [18,35].

Accordingly, in this study, we found a frequency of TDs twice as high in patients with CD compared to subjects without CD (25.0% vs. 13.6%), with the most represented disorders being HT that is associated with a hypofunction of the gland. In a previous study performed in the same geographic area, AIDs occurred more frequently in celiac patients than in controls (35.3% vs. 15.2%), with an OR of having at least one additional AID of 3.31 after adjusting for all covariates [15], and similar to our findings, HT was the most prevalent AITD (24.3% vs. 10%; *p* < 0.0001). In a large sample of the general population of Southern Sardinia, including a total of 25,885 adult subjects, the prevalence of autoimmune thyroiditis, diagnosed by a specialist, was the highest among 12 AIDs [26].

Moreover, the present study revealed that, in addition to the expected increased frequency of AITDs in CD, non-AITDs were also increased, in particular in patients requiring thyroidectomy. We can hypothesize that, especially in the past, when CD was considered rare, patients were subjected to strict follow-up, mostly for autoimmune disorders screening, which might have favored an overestimation of AITDs. This (possible) overestimation of TDs due to thyroid screening by ultrasound scan was a very popular practice until 2014, when Lee and Shin showed that screening for thyroid cancer lead to an overdiagnosis without benefit on mortality rates, despite additional medical costs and psychological stress on the population [36].

In a meta-analysis published a few years ago, including a total of 15,629 CD cases and 79,342 controls, the overall prevalence of TDs in patients with CD was significantly increased compared with the control group (OR 3.08, *p* < 0.001) [24]. Contrary to Ventura’s hypothesis, in this meta-analysis, a significant difference in the ORs between the gluten-treated and untreated groups was not observed (OR 1.08, *p* = 0.786) [24]. Unfortunately, in our study, data about treated and untreated CD patients were unavailable, making comparison unfeasible. Similar results were obtained from a multicenter study conducted in Italy [37]. Thyroid disorders were detected three-fold more frequently in patients with CD than in controls (*p* < 0.0005), with autoimmune hypothyroidism being more common (12.9%) than non-autoimmune TDs (8.7%) [37]. As seen in our cohort, hyperthyroidism was equally represented in cases and controls. However, autoimmune TDs with euthyroidism were present in 16.2% of CD patients in the multicenter study [37] and in 2.7% of our cohort. The large discrepancy between the prevalence of autoimmune TDs without functional disorders may be the result of the different study designs. In the multicenter study, subjects went through a full assessment of thyroid function via the measurement of the levels of thyroid hormones, thyroperoxidase, and thyroid microsome Abs regardless of symptoms [37]. In our study, CD patients underwent an endocrinological visit guided by symptoms, which may explain the low prevalence of autoimmune TDs with euthyroidism. Nonetheless, autoimmune TDs with euthyroidism were statistically more frequent in CD patients than in controls (2.7% vs. 1.1%, *p* < 0.001).

In 2009, Meloni et al., in a retrospective study, analyzed the prevalence of AITDs in Sardinian CD children and the effects of a GFD on thyroid function in 324 children matched with schoolchildren (*n* = 8040) from the same geographic area who had been previously evaluated for AITDs [29]. Among CD patients, AITDs were detected in 10.5%, and in 2.9% of controls, more frequently in females than males, with a ratio of 4.5:1 [29]. More specifically, among AITDs, the absence of functional alterations was more frequent than hypothyroidism, in contrast to our study cohort. This contrast may be justified by the different ages of the two populations studied (children vs. adults). On the other hand, the prevalence of CD in AITD patients, in a study conducted in the same geographical area, was four-fold greater than that observed in the general population (4.37 vs. 1.06%, *p* < 0.0001) [11].

As suggested by Fasano et al., it is possible that, besides the shared genetic background responsible for CD and the co-expressed Abs, the persistence of gluten exposure in untreated CD subjects, or accidentally ingested gluten in CD patients on a GFD, might alter the intestinal barrier and, in turn, its function in preserving the systemic immune response [38]. Accordingly, CD patients, with and without poly-autoimmune disease, displayed significant differences in the gut microbiota composition [39,40]. Of note, the occurrence of CD is more likely in patients with type 1 diabetes, thyroiditis, and psoriasis [41]. The gluten-induced immune reactivity may have important clinical implications because, when the circle of gluten exposure, intestinal mucosa alterations, gut microbiota unbalance, and autoimmune predisposing genes is interrupted, the occurrence of additional AIDs could be stopped [42]. In fact, the association between gluten exposure and autoimmune disorders might be due to molecular mimicry. The production of neo-epitopes might also enhance the development of other autoimmune conditions in CD [43]; in fact, intermolecular T-cell epitope spreading has been demonstrated in animal models of autoimmune diseases [44]. The hypothesis that the gluten load might be involved in the development of autoimmune diseases in CD, apart from Ventura et al., was corroborated by other investigators. For example, Di Mario et al. observed that 27% of children with untreated CD showed anti-insulin antibodies, compared with 20% of treated celiac children and none of the controls [45]. Similarly, antibodies against thyroid peroxidase and other endocrine-related autoantibodies were present in 53% of adolescents with untreated CD and 20% of non-GFD adolescents. Moreover, the titers were higher in untreated disease [46].

### Limitations

The main limitation of our study is the retrospective design, with cases (CD patients) included in the study after outcomes (TDs) have already occurred in some of them, and the consequent disadvantage is that the quality of the data is inferior to that of a prospective study. For example, a number of subjects with HT in euthyroidism could have been missed, especially among controls, considering that CD patients are more prone to undergoing specialist visits more frequently than the general population. Nonetheless, AITD in euthyroidism was statistically more common in cases than in controls. Moreover, additional factors, able to eventually influence the outcome, such as accidental or volunteer exposure to gluten, were not collected. However, the strengths of our study are the homogeneity of the genetic background of the analyzed cohort and the large sample size, which allow for reliable estimations of the risk of TDs, and more specifically AITDs, in patients with CD.

## 5. Conclusions

In this study, we found a clinically important association between TDs and CD. Based on these results, thyroid function should be checked routinely in celiac patients at presentation and, if found to be normal, re-checked during follow-up.

## Figures and Tables

**Table 1:** Descriptive statistics in 8489 study participants according to thyroid disorders.

Variables	No Thyroid Disease (*n* = 7312)	Thyroid Disease (*n* = 1177)
Sex, *n* (%)		
Female	4437 (80.8)	1053 (19.2) **
Male	2875 (95.9)	124 (4.1)
Age, mean ± SD	51.67 ± 17.52	54.78 ± 15.23
Area, *n* (%)		
Urban	3705 (86.5)	580 (13.5)
Rural	3607 (85.8)	597 (14.2)
Occupation, *n* (%)		
I	593 (85.7)	103 (14.3)
II	1725 (86.3)	281 (13.7)
III	2296 (90.9)	237 (9.1)
IV	2698 (83.0)	556 (17.0) *
BMI, *n* (%)		
18–24 kg/m^2^	4197 (86.6)	651 (13.4)
25–29 kg/m^2^	2266 (85.6)	380 (14.4)
≥30 kg/m^2^	849 (85.3)	146 (14.7)
Smoke, *n* (%)		
No	3876 (85.5)	655 (14.5)
Yes	3436 (86.8)	522 (13.2)
Celiac disease, *n* (%)		
No	6851 (87.1)	1015 (12.9)
Yes	461 (74.0)	162 (26.0) **

* *p* < 0.05 ** *p* < 0.001.

**Table 2:** Frequency of thyroid disorders in adult patients according to presence of celiac disease.

Variables	No Celiac Disease (*n* = 7866)	Celiac Disease (*n* = 623)
Thyroid disorders total no. (%)		
No Thyroid Disorders	6851 (93.7)	461 (74.0)
Autoimmune Thyroid Disorders	594 (7.5)	96 (15.4) **
*Hashimoto’s in euthyroidism*	84 (1.1)	17 (2.7) **
*Hashimoto’s with hypofunction ^§^*	443 (5.6)	71 (11.4) **
*Graves’ disease*	67 (0.8)	8 (1.3)
No Autoimmune Thyroid Disorders	421 (5.1)	66 (10.6) **
*Thyroid nodules/goiter*	141 (1.8)	6 (0.9)
*Iatrogenic thyroid hypo/hyperfunction*	25 (0.3)	1 (0.2)
*Thyroidectomy ^#^*	255 (3.2)	59 (9.5) **

** *p* < 0.001, ^§^ matched with treatment, ^#^ for nodules, goiter, and/or cancer.

**Table 3:** Multiple logistic regression for autoimmune thyroid disease (matched with treatment).

Variables	Unadjusted OR (95% CI)	Adjusted OR (95% CI)
*Sex*		
Male	Ref.	Ref.
Female	6.031 (4.663–7.800) **	5.855 (4.434–7.731) **
*Age*	1.006 (1.001–1.010) *	1.012 (1.007–1.017) **
*Area*		
Urban	Ref.	Ref.
Rural	0.997 (0.856–1.161)	1.100 (0.937–1.291)
*Occupation*		
I	Ref.	Ref.
II	0.879 (0.641–1.205)	0.999 (0.723–1.379)
III	0.583 (0.423–0.804) **	0.837 (0.600–1.167)
IV	1.311 (0.980–1.754)	1.028 (0.763–1.387)
*Body mass index*	0.995 (0.976–1.014)	1.004 (0.984–1.024)
*Smoke*		
No	Ref.	Ref.
Yes	0.978 (0.839–1.140)	1.135 (0.932–1.382)
*Celiac disease*		
No	Ref.	Ref.
Yes	2.382 (1.901–2.983) **	2.387 (1.857–3.068) **

* *p* < 0.05, ** *p* < 0.001

## Data Availability

Data will be made available upon specific request to the corresponding author.
